# Long-term desensitization for ABO-incompatible living related kidney transplantation recipients with high refractory and rebound anti-blood type antibody: case report

**DOI:** 10.1186/s12882-018-1053-8

**Published:** 2018-10-05

**Authors:** Hiroaki Nishimura, Yasutoshi Yamada, Satoshi Hisano, Akihiko Mitsuke, Syuichi Tatarano, Takenari Gotanda, Hiroshi Hayami, Masayuki Nakagawa, Hideki Enokida

**Affiliations:** 1 Department of Urology, Graduate School of Medical and Dental Sciences, Kagoshima University, Kagoshima, Japan; 2 Department of Pathology, Fukuoka University school of Medicine, Fukuoka, Japan; 3 Department of Urology, Kagoshima City Hospital, Kagoshima, Japan

**Keywords:** ABO-incompatible living related kidney transplantation, Anti-blood type antibody, B-cell immunity, Mycophenolate mofetil

## Abstract

**Background:**

ABO-incompatible living related kidney transplantation (ABO-iLKT) has increased the possibilities for kidney transplantation in patients with end stage renal disease. Due to advancements in immunosuppressive agents and the identification of immunological conditions following ABO-iLKT, this transplantation technique has achieved the same success rate as ABO-compatible LKT. However, some patients continue to generate anti-blood type antibodies, despite conventional immunosuppressant treatment.

**Case presentation:**

A 60-year-old man was referred to our hospital for kidney transplantation. The proposed transplant was ABO incompatible, from a donor with blood-type A to a recipient with blood-type O. The recipient’s anti-A blood-type IgG antibody titer was measured at 4096-fold dilution. Following desensitization therapy, including mycophenolate mofetil (MMF) 750 mg/day for 3 months, intravenous Rituximab 200 mg, and two sessions of double filtration plasmapheresis, the anti-A blood-type IgG antibody titer decreased to only 516-fold dilution and did not meet our target of less than 128-fold dilution. MMF was thus continued for an additional 4 months and four additional sessions of plasmapheresis were undertaken. Following these interventions, antibody titers decreased to 128-fold dilution and ABO-iLKT was performed. Following transplant, antibody-mediated rejection was not observed and renal function was preserved. However, a post-operative renal biopsy 1.5 months later showed evidence of T-cell-mediated rejection IB. The patient was treated with steroids, with no increase in serum creatinine.

**Conclusion:**

Our findings suggest that the long-term single MMF desensitization therapy could be a suitable option for ABO-iLKT with high refractory and rebound anti-blood type antibody. Further studies are required to establish the optimal immunosuppression regimen to control B cell- mediated immunity in ABO-iLKT.

## Background

Kidney transplantation is the most effective renal replacement therapy for improving mortality and quality of life [1]. However, while the number of patients waiting for a donor kidney is increasing, there is a shortage of organ transplantation donors [2]. One strategy to address this problem is ABO-incompatible living related kidney transplantation (ABO-iLKT).

ABO-iLKT has the potential to expand the opportunities for kidney transplantation. This transplantation method has been performed since 1982, and Opelz et al. reported on 1420 patients who received ABO-incompatible kidney grafts between 2005 and 2012 [3]. ABO-iLKT has been successful, in part, because of the identification of immunological mechanisms following the procedure, including accommodation, humoral rejection, and cellular rejection [4, 5]. The maintenance of a vascularized graft despite the presence of anti-blood-group antibodies is termed ‘accommodation’ [4]. Accommodation can be established with pre- and post-transplant conditioning regimens. Despite the development of modern conditioning treatments, some patient populations continue to have a high risk of transplant rejection.

Our report describes the clinical course of a patient undergoing ABO-iLKT with refractory high-titer (anti-A blood-type IgG antibody titer: 4096-fold dilution) and rebound anti-blood type antibody. We discuss the influence of long-term desensitization therapy on kidney transplantation in similar high-risk patients.

## Case presentation

A 60-year-old man was referred to our hospital for kidney transplantation. His wife, a 59-year-old woman, volunteered to donate her kidney to him when he started hemodialysis at age 59. The proposed transplant was ABO incompatible, from a donor with blood-type A to a recipient with blood-type O, and the recipient’s anti-A blood-type IgG antibody titer was measured at 4096-fold dilution.

Preoperative testing included HLA-DNA typing, which revealed a mismatch in 6 antigens. Initial flow cytometric crossmatch testing (FCXM) was negative. Moreover, the flow cytometric panel reactive antibody (Flow PRA) screening test was negative for human leukocyte antigen (HLA) class I and class II. Single antigen testing was also negative.

**Fig. 1:**
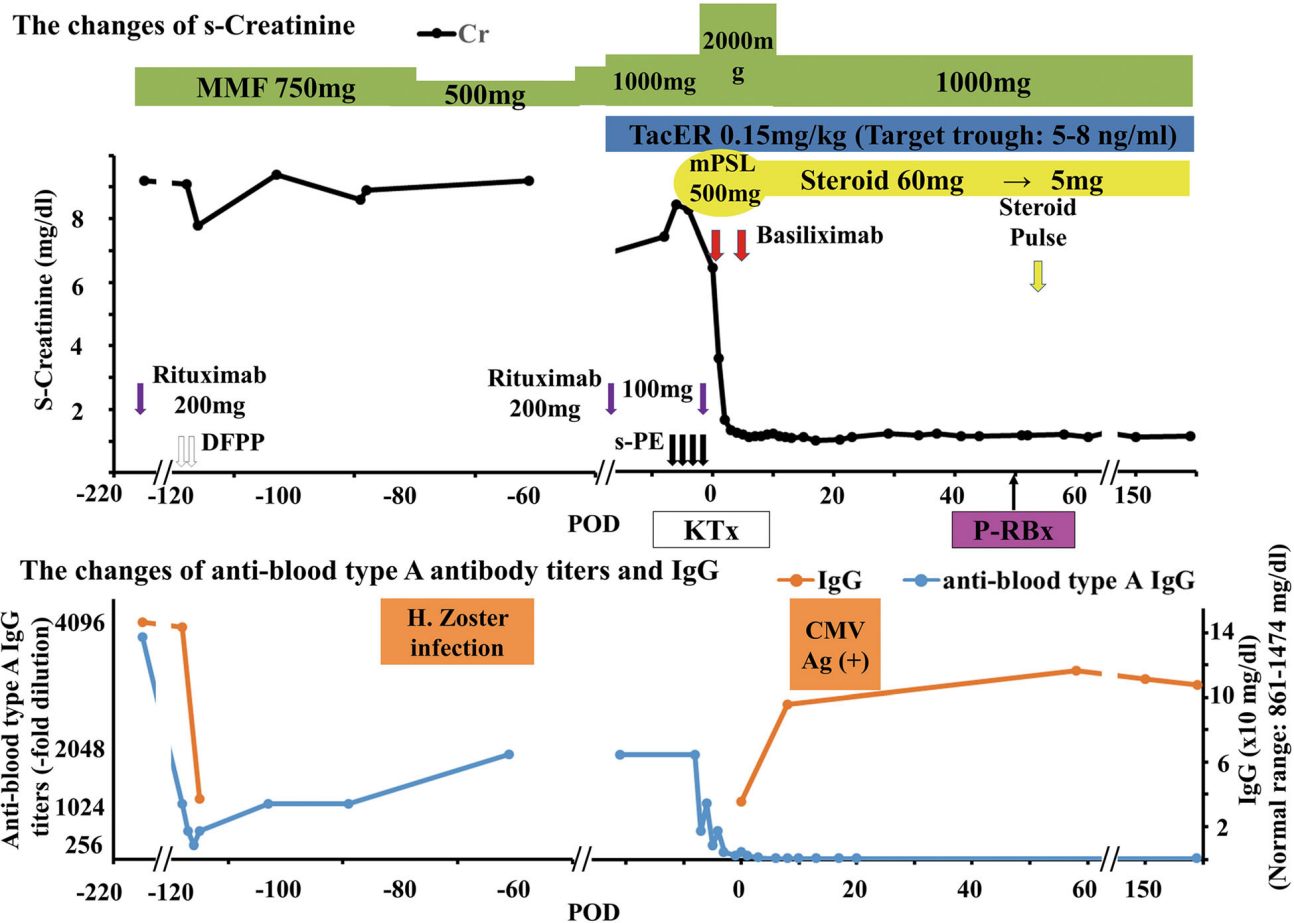
Patient’s clinical course and laboratory data: serum creatinine, anti-blood type A antibody titers, and IgG

Three months prior to surgery, mycophenolate mofetil (MMF) 750 mg/day was initiated and the anti-CD20 monoclonal antibody Rituximab (200 mg) was administered according to our pre-transplantation regimen (Fig. 1). Following 3 months of desensitization therapy, the patient underwent two sessions of double filtration plasmapheresis (DFPP).

Anti-blood type antibody titers (IgG/IgM) were then assayed using the column agglutination technology (gel microcolumn) method (Bio-Rad®, Japan). Our target antibody titer level was < 128-fold dilution; however, the anti-A blood-type IgG antibody titer decreased to only 512-fold dilution (Table 1). In addition, serum IgG before the induction of DFPP was 1428 mg/dl. The high titer state following plasmapheresis was considered “refractory rebound”, and the planned transplant was postponed in order to resume desensitization therapy (MMF 750 mg/day). Four months following the initial start of desensitization therapy (MMF), the anti-A blood-type antibody level rebounded to 1024-fold dilution.

**Table 1:** anti-blood type antibody changing during peri-DFPP treatments

	1st DFPP		2nd DFPP		
	(Day 0)		(Day 1)		(Day 2)
	Pre	Post	Pre	Post	
anti-blood type IgG titer (−fold dilution)	1024	512	512	256	512
anti-blood type IgM titer (−fold dilution)	128	32	32	16	32

*DFPP* Double filtration plasmapheresis

Shortly after the re-initiation of desensitization therapy (150 days from the initial start of therapy), the patient developed herpes zoster infection. He was treated with anti-viral medication and the MMF dose was reduced from 750 mg/day to 500 mg/day. After 1 month, the MMF dose was increased back to 750 mg/day.

Transplantation was rescheduled to occur 210 days from the initiation of MMF. The pre-transplantation regimen was as follows. Rituximab was administered at 200 mg and 100 mg at 21 days and 1 day before transplant, respectively. Twelve days prior to surgery, the dose of MMF was increased to 1000 mg/day (At 11 days prior to surgery, serum MMF Area Under the Curve (AUC)_0–12_ was 35.6 ng/ml.). The initial dose of extended-release tacrolimus (TacER) (0.15 mg/kg/day) was administered 13 days prior to transplantation and the dose was adjusted based on serum concentration. Because the initial DFPP sessions did not decrease the anti-A blood-type antibody titers, 4 sessions of selective plasma exchange (PE) were used to remove the anti-blood type antibody. With these interventions, the anti-A blood-type IgG antibody decreased to 128-fold dilution and the serum IgG level decreased to 357 mg/dl on the day of transplant (Table 2).

**Table 2:** anti-blood type antibody changing during peri-sPE treatments

	1st sPE		2nd sPE		3rd sPE		4th sPE		Operation day
	(Day 0)		(Day 2)		(Day 4)		(Day 7)		(Day 8)
	Pre	Post	Pre	Post	Pre	Post	Pre	Post	
anti-blood type IgG titer (−fold dilution)	2048	N/A	1024	256	512	128	64	64	128
anti-blood type IgM titer (−fold dilution)	128	N/A	N/A	N/A	64	32	32	16	32

*sPE* selective Plasma exchange

**Fig. 2:**
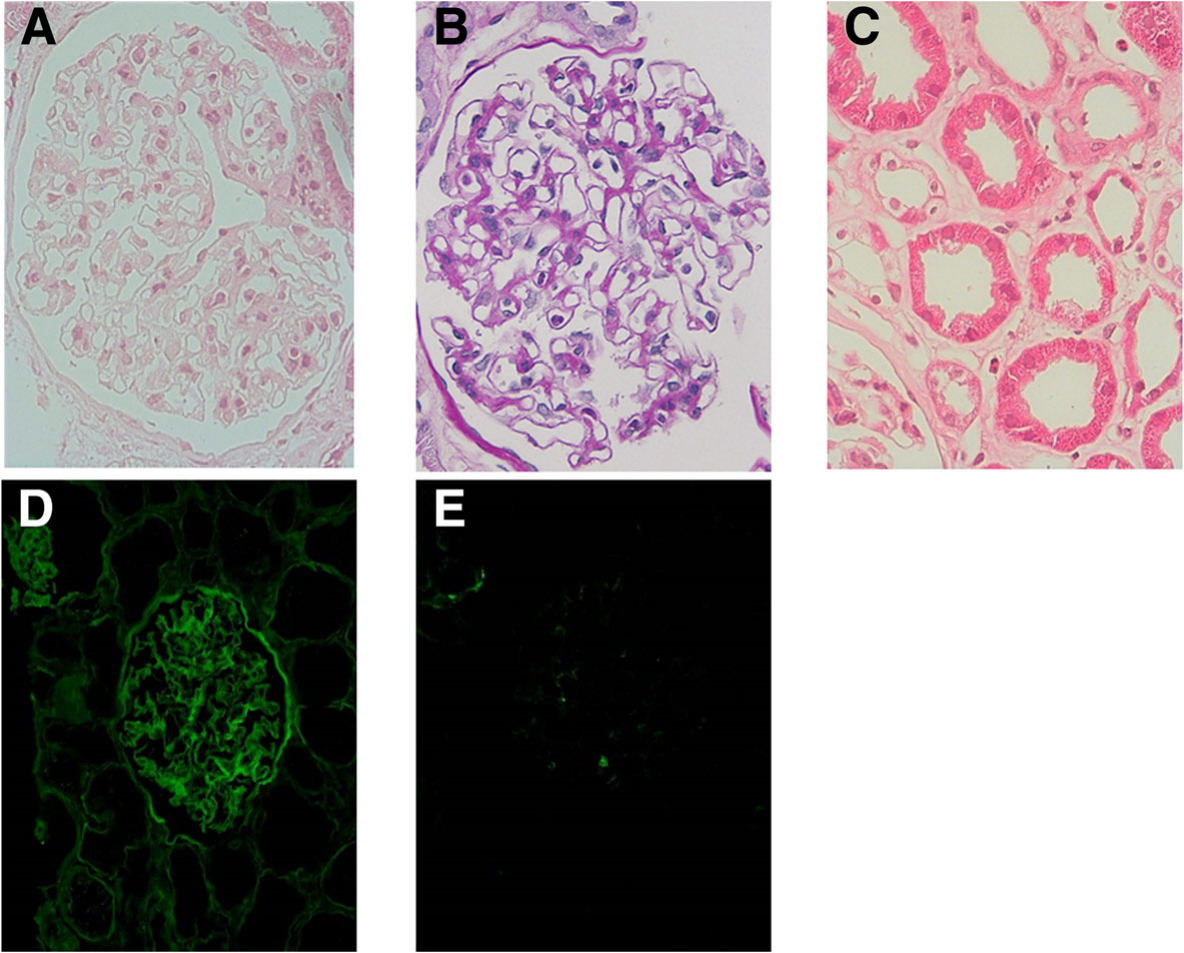
Histology of 1 h-biopsy. Glomeruli were normal. Glomerular basement membrane was unremakable. Tubulointerstitium was diffusely edematous. Arteries were unremarkable. **a, c** Hematoxylin and eosin (H.E) staining × 400, (**b**): Periodic acid-Schiff (PAS) × 400). Immunohistochemistry showed limited mesangial IgG deposits (**d**) and no IgM deposits (**e**). C4d immunofluorescence result was negative (not shown)

The renal graft was transplanted into the right iliac fossa without incident. Subsequently, the graft became pink and urine was produced immediately. The post-transplant induction immunosuppression protocol consisted of TacER, MMF 2000 mg/day, basiliximab 20 mg administered on postoperative day (POD) 0 and 4, and systemic steroids starting on POD 0. A graft biopsy performed 1 h after reperfusion demonstrated no evidence of hyperacute rejection (Fig. 2). The serum creatinine (s-Cr) level began to decrease immediately. On POD 6, the s-Cr level was 1.5 mg/dl, and anti-A blood-type IgG and IgM antibodies were measured at 16-fold and 4-fold dilutions, respectively. The antibody titer levels remained at these levels throughout the post-operative course. However, serum IgG increased to 957 mg/dl. On POD 12, cytomegalovirus (CMV) antigenemia was diagnosed. The antiviral medication baragancyclovir was initiated and the dose of MMF was decreased to 1000 mg/day. No further post-operative complications were observed.

**Fig. 3:**
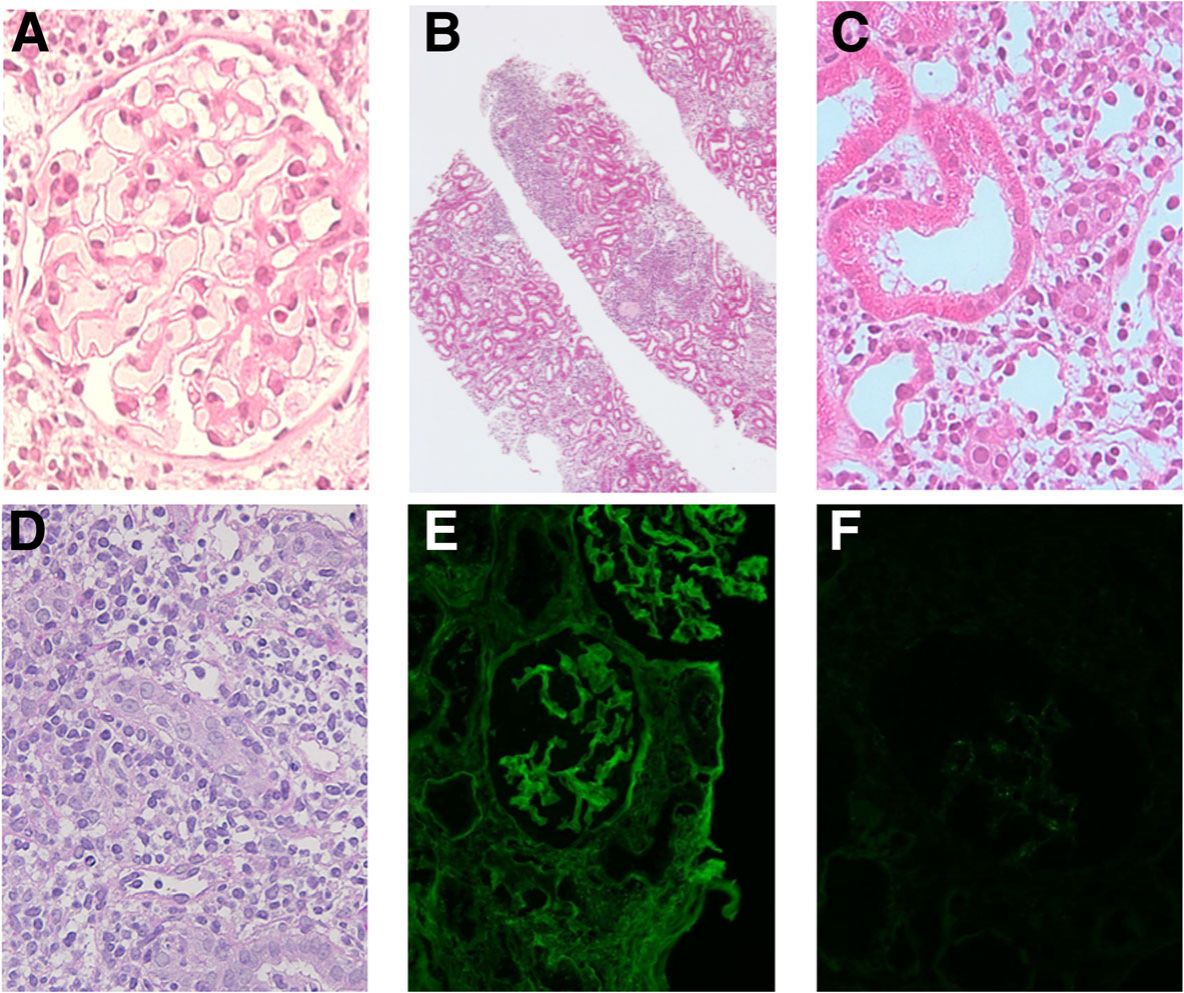
Histology of biopsy 1.5 months after transplantation. Glomeruli were normal. Patchy and moderate-to-severe infiltration of mononuclear cells in the interstitium and focal infiltration of mononuclear cells in the proximal tubular epithelium were observed. **a**, **c** H.E × 400, (**b**, **d**) PAS × 40, × 400). Immunohistochemistry demonstrated limited mesangial IgM deposits (**f**) with no IgG deposition in the glomeruli (**e**). C4d immunofluorescence result was negative (not shown)

The sCr fluctuated between 1.29 and 1.42 mg/dl during the 1.5 months after ABO-iLKT. A protocol biopsy was performed on POD 50. The histopathological examination revealed i) acute T cell-mediated rejection IB, and ii) no evidence of acute antibody-mediated rejection (Fig. 3). Steroid pulse therapy (500 mg for 3 consecutive days) was administered.

At the time of 18 months after ABO-iLKT, the sCr level was between 1.30 and 1.49 mg/dl (estimated glomerular filtration rate (eGFR)-calculation of 38.0–40.8 ml/min/1.73m^2^). Urinalysis showed urine albumin of 30–80 mg/L, urine red blood cells of 0–1 /high power field, urine white blood cells 0–1 /high power field and no granular casts. His blood pressure was maintained as 132/80–138/88 mmHg on Amlodipine 5 mg once daily. Anti-A blood-type IgG and IgM antibody titers were stable at 16-fold and 8-fold dilutions, respectively. Serum IgG was preserved within the normal range. The patient was maintained on triple immunosuppression therapy consisting of TacER, MMF 1000 mg/day, and steroid 5 mg/day.

## Discussion

Initial attempts at ABO-iLKT were associated with high rates of early graft loss due to acute antibody-mediated rejection [6]. But recently, Aikawa et al. reported that there were no differences in patient or graft survival rates between ABO-iLKT versus ABO-compatible transplantation, based on data from 2434 ABO-iLKTs collected from 120 Japanese kidney transplant centers [7]. Other studies have suggested that the occurrence of antibody- mediated rejection following ABO-iLKT is higher in cases of refractory reappearance of anti-blood type antibody than in cases of slight reappearance [8]. Even now, when ABO-iLKT routinely achieves long-term success, the evaluation and treatment of anti-blood type antibodies remain integral to transplant survival.

The levels of anti-blood type antibodies that are safety tolerated in successful ABO-iLKT appear to differ between transplant laboratories [7–10] due to the use of different measurement techniques [11] and therapeutic methods. Removing anti-blood type antibodies by blood purification methods such as PE or plasmapheresis, splenectomy, and anti-B-cell immunosuppressants has been shown to prevent acute antibody-mediated rejection. Flint et al. demonstrated that ABO-iLKT with refractory anti-blood type antibodies may be performed without prolonged preoperative immunosuppression treatments, although the patients in this series developed antibody-mediated rejection despite frequently preoperative PEs [12]. Hence, blocking anti-blood type antibody production, rather than removing anti-blood type antibodies, appears to be extremely important for preventing antibody- mediated rejection.

Rituximab is an anti-B-cell immunosuppressant that depletes B cells in the peripheral blood during perioperative conditioning of ABO-iLKT. This effect is comparable to splenectomy and has been successfully used in patients with high levels of anti-blood type antibodies [13]. Other desensitization treatments include Bortezomibe and Eculizumab [14, 15], but the sites of action are limited with these agents. It has been recommended that Bortezomibe and Eculizumab be used in conjunction with rituximab and blood purification following conventional desensitization treatments. Recently, multiple centers in Korea and Japan reported good outcomes in ABO-iLKT without splenectomy using Rituximab at 200 mg/person or 100 mg/m^2^ [16]. These studies additionally demonstrated B-cell recovery at 3–6 months after 15 mg/m^2^ Rituximab administration, and PE decreased the therapeutic effect. Consequently, we chose to administer additional Rituximab before transplantation in our study.

Rituximab targets mature B cell subsets but has no effect on plasma cells in the spleen or in the secondary lymphoid organs [8]. For this reason, most conventional preconditioning treatments cannot prevent antibody-mediated rejection in rebound cases. Some studies have demonstrated that several months of cyclophosphamide treatment lowers anti-blood type antibody and anti-HLA antibody titers to optimal transplantation levels [17, 18]. Hence, a long-term desensitization protocol might have higher efficacy in immunological high-risk cases. We used long-term single MMF desensitization. MMF blocks expansion of both naïve and memory B cells and prevents plasma cell differentiation, leading to control of humoral immunity [19]. We continued desensitization therapy longer than conventional preoperative conditioning treatments [4, 9, 13] and the treatment protocol in our hospital during the last 5 years (Table 3). Fassbinder et al. reported significant decreases in plasma cell counts 16 weeks after induction therapy with MMF and persisting to 31 weeks [20]. Based on this data and our own findings, we speculated that long-term single MMF desensitization lasting four to 7 months would produce humoral immunity in refractory cases, leading to to optimal conditions for successful ABO-iLKT.

**Table 3:** Immunosuppression-regimens by antiblood type IgG and/or IgM titer range and outcome of ABO incompatible transplantation

Group 1: 13 cases with antiblood type IgG and/or IgM titer range of 2 to 256-fold dilution	
Regimen	
MMF^a^	250–500 mg bid commencing 12–25 days pretransplant, increase 1000 mg bid at time of transplant. Taper to a total dose of 1500 mg/day by weeks 2, then 1000 mg/day by weeks 24.
TacER^b^	0.1 mg/kg/day commencing 7–21 days pretransplant, adjusted to levels of 5 ng/ml for these days (then titrated to 5–7 ng/ml 2 days pretansplant), 5–7 ng/ml 2 weeks posttransplant, 4.5–6.5 ng/ml 3–12 weeks, 4–5.5 ng/ml 13 weeks
Steroids	Methylprednisolone 500 mg at transplant, then 60 mg/day weaning 5 mg by 21 days posttransplant
Basiliximab	20 mg day 0 and day 4
Rituximab	200 mg 21 days pretransplant
Plasma exchange^c^	1–4 times pretransplant
Outcome	All 13 cases are alive and their grafts are functioning. Protocol and episode biopsies revealed one antibody mediated rejection due to DSA and one borderline change. There were no evidence of rejection in other 11 cases.
Group 2: Two cases with antiblood type IgG titer of 1024-fold dilution and IgM titer of 64 or 128-fold	
Regimen	
MMF^a^	500 mg bid commencing 118 or 63 days pretransplant, increase 1000 mg bid at time of transplant. Taper to a total dose of 1500 mg/day by weeks 2, then 1000 mg/day by weeks 24.
TacER^b^	0.1 mg/kg/day commencing 11 or 30 days pretransplant, adjusted to levels of 5 ng/ml for these days (then titrated to 5–7 ng/ml 2 days pretansplant), 5–7 ng/ml 2 weeks posttransplant, 4.5–6.5 ng/ml 3–12 weeks, 4–5.5 ng/ml 13 weeks
Steroids	Methylprednisolone 500 mg at transplant, then 60 mg/day weaning 5 mg by 21 days posttransplant
Basiliximab	20 mg day 0 and day 4
Rituximab	200 mg 21 days and 100 mg 1 day pretransplant
Plasma exchange^c^	7 or 12 times pretransplant
Outcome	All two cases are alive and their grafts are functioning. Protocol biopsies revealed no evidence of rejection, respectively.
Group 3: Five cases with antiblood type IgG and/or IgM titer of 4 to16-fold dilution	
Regimen	
MMF^a^	250–500 mg bid commencing 7–2 days pretransplant, increase 1000 mg bid at time of transplant. Taper to a total dose of 1500 mg/day by weeks 2, then 1000 mg/day by weeks 24.
TacER^b^	0.1 mg/kg/day commencing 7–15 days pretransplant, adjusted to levels of 5 ng/ml for these days (then titrated to 5–7 ng/ml 2 days pretansplant), 5–7 ng/ml 2 weeks posttransplant, 4.5–6.5 ng/ml 3–12 weeks, 4–5.5 ng/ml 13 weeks
Steroids	Methylprednisolone 500 mg at transplant, then 60 mg/day weaning 5 mg by 21 days posttransplant
Basiliximab	20 mg day 0 and day 4
Rituximab	200 mg 21 days pretransplant
Plasma exchange^c^	Plasma exchange has not be undergone during their clinical courses.
Outcome	All five cases are alive and their grafts are functioning. Protocol biopsies revealed no evidence of rejection in all cases.

^a^*MMF* Mycophenolate mofetil, ^b^*TacER* extended-release tacrolimus, ^c^Plasma echange method (: DFPP, sPE, PE), exchange volume and exchange contents were adjusted to anti-ABO antibody titer or allergic tendency

We conducted therapeutic drug monitoring (TDM) of mycophenolic acid (MPA) throughout the perioperative period. The target therapeutic window of the MPA abbreviated area under the blood concentration-time curve (AUC) from 0 to 12 h (AUC_0–12_) is recommended to be 30–60 mg·h/L [21]. Higher MPA may be required for patients with high immunologic risk, but we presumed that 30–60 mg·h/L of the MPA AUC_0–12_ during long-term MMF administration would be adequate for this study.

Since both T cell- and B cell-mediated immunity influence clinical outcomes in ABO-iLKT, cellular rejection should also be addressed following transplantation [5]. Prior studies have reported no significant differences in the incidence of acute T cell-mediated rejection between ABO-iLKT and ABO-compatible LKT [22]. The side effects of MMF treatment include gastrointestinal intolerance, hematologic complications, and infections [23]. During our long-term desensitization protocol, we observed viral infections including herpes zoster and CMV infections. These infections were treated with anti-viral medications and a dose reduction of MMF, and they did not lead to serious illness. However, the dose reduction of MMF might have influenced the occurrence of subsequent acute T cell-mediated rejection. Several studies have assessed the benefit of TDM of MPA, although it is uncertain whether monitoring reduces the incidence of infection [24]. To prevent infection while maintaining targeted immunosuppressant levels, we are currently administering prophylactic antiviral medication during long-term desensitization and in the post-transplant period in refractory cases.

One option for patients with refractory rebound anti-blood type antibodies is Kidney-paired donation. de Klerk et al. reported an excellent 5-year graft survival in ABO-blood-group incompatible donor-recipient pairs from the Netherlands National Living Donor Kidney Exchange Program [25]. On the other hand, Lonze et al. demonstrated that the simultaneous expansion of both effective desensitization protocols and ABO-blood-group incompatible donor exchange provided the benefit to recipients with both ABO i- and HLA i-LKT in cases of high sensitization to donor’s HLA [26]. Kidney-paired donation could identify the donor with weakest DSA-strength, and the patients’ survival would be better than those waiting on dialysis for a compatible kidney. Certainly, the recipients with both ABO i- and HLA i-LKT trended towards antibody-mediated rejection compared to those with ABO i-LKT alone [26]. These facts indicate that the optimal desensitization will also affect the diverse patients in the field of Kidney-paired donation in the future..

Furthermore, immunosuppressant protocols would be a concern with long-term desensitization treatment. Inui et al. demonstrated that a quadruple immunosuppressant desensitization protocol (Cyclosporine, MMF, methylprednisolone, and Rituximab) in addition to blood-purification and intravenous immunoglobulin was able to maintain anti-blood type antibodies titer of a 100-fold or less during 4 months without complications [27]. Thus, the long-term administration of multiple agents might be useful with excellent results. However, in the current case, we adopted a single agent (MMF) for a long-term immunosuppressant desensitization because adverse events of multiple agents for long-term administration of have not been elucidate. In another aspect, our method could be one of effective alternate strategies for patients who are unfit for the long-term desensitization with multiple agents.

## Conclusion

Our study highlights the effectiveness of long-term desensitization methods for refractory and rebound anti-blood type antibodies in ABO-iLKT. However, there are no randomized trials comparing the effectiveness of various desensitization protocols. Further studies are required to establish the optimal immunosuppression regimen to control B cell- mediated immunity in ABO-iLKT.

## Abbreviations

ABO-iLKT: ABO-incompatible living related kidney transplantation
AUC: Area under the curve
CMV: Cytomegalovirus
Cr: Serum creatinine
DFPP: Double filtration plasmapheresis
FCXM: Flow cytometric crossmatch test
Flow PRA: Flow cytometric panel reactive antibody
HLA: Human leukocyte antigen
MMF: Mycophenolate mofetil
MPA: Mycophenolic acid
PE: Plasma exchange
TDM: Therapeutic drug monitoring

## References

[1] Wolfe RA, Ashby VB, Milford EL, Ojo AO, Ettenger RE, Agodoa LY (1999). Comparison of mortality in all patients on dialysis, patients on dialysis awiting transplantation, and recipients of a first cadaveric transplant. N Engl J Med.

[2] UNOS Transplant Trends. 1999. https://unos.org/. Accessed 3 Dec 2012.

[3] Opelz G, Morath C, Süsal C, Tran TH, Zeier M, Döhler B (2015). Three-year outcome following 1420 ABO-incompatible living-donor kidney transplants performed after ABO antibody reduction: result from101 centers. Transplantation.

[4] Takahashi K, Saito K, Takahara K, Okuyama A, Tanabe K, Toma H (2004). The Japanese ABO-incompatible kidney transplantation committee. Excellent long-term outcome of ABO-incompatible living donor kidney transplantation in Japan. Am J Transplant.

[5] Takahashi K (2007). Recent findings in ABO-incompatible kidney transplantation: classification and therapeutic strategy for acute antibody-mediated rejection due to ABO-blood-group-related antigens during the critical period preceding the establishment of accommodation. Clin Exp Nephrol.

[6] Rydberg L (2001). ABO-incompatibility in solid organ transplantation. Transfus Med.

[7] Aikawa A, Sito K, Takahashi K (2015). Trends in ABO-incompatible kidney transplantation. Exp Clin Transplant.

[8] Ishida H, Kondo T, Shimizu T, Nozaki T, Tanabe K (2015). Postoperative rebound of antiblood type antibodies and antibody-mediated rejection after ABO-incompatible living-related kidney transplantation. Transpl Int.

[9] Montgomery RA, Locke JE, King KE, Segev DL, Warren DS, Kraus ES (2009). ABO incompatible renal transplantation: a paradigm ready for broad implementation. Transplantation.

[10] Aikawa A, Kawamura T, Shishido S, Saito K, Takahashi K, ABO-incompatible transplantation committee members (2014). ABO-incompatible living-donor pediatric kidney transplantation in Japan. Clinics (Sao Paulo).

[11] Shirey RS, Cai W, Montgomery RA, Chhibber V, Ness PM, King KE (2010). Streamlining ABO antibody titrations for monitoring ABO-incompatible kidney transplants. Transfusion.

[12] Flint SM, Walker RG, Hogan C, Haeusler HN, Robertson A, Francis DMA (2011). Successful ABO-incompatible kidney transplantation with antibody removal and standard immunosuppression. Am J Transplant.

[13] Uchida J, Kuwabara N, Machida Y, Iwai T, Nagamura N, Kumada N (2012). Excellent outcomes of ABO-incompatible kidney transplantation: a single center experience. Transplant Proc.

[14] Wong NL, O’connell P, Chapman JR, Nankivell B, Kable K, Webster AC (2015). Bortezomib in ABO-incompatible kidney transplant desensitization: a case report. Nephrology.

[15] Stewart ZA, Collins TE, Schlueter AJ, Raife TI, Holanda DG, Nair R (2012). Case report: Eculizumab rescue of severe accelerated antibody-mediated rejection after ABO-incompatible kidney transplant. Transplant Proc.

[16] Koo TY, Yang J (2015). Current progress in ABO-incompatible kidney transplantation. Kidney Res Clin Pract.

[17] Cohney SJ, Walker RG, Haeusler MN, Francis DM, Hogan CJ (2007). Blood group incompatibility in kidney transplantation: definitely time to re-examine. MJA.

[18] John R, Lietz K, Burke E, Ankersmit J, Mancini D, Suciu-Foca N (1999). Intravenous immunoglobulin reduces anti-HLA alloreacten waiting time to cardiac transplantation in highly sensitized left ventricular assist device recipients. Circulation.

[19] Karnell JL, Karnell FG, Stephens GL, Rajan B, Morehouse C, Li Y (2011). Mycophenolic acid differentially impacts B cell function depending on the stage of differentiation. J Immunol.

[20] Fassibinder T, Saunders U, Mickholz E, Jung E, Becker H, Schlüter B (2015). Differential effects of cyclophosphamide and mycophenolate mofetil on cellular and serological parameters in patients with systemic lupus erythematosus. Arthritis Res ther.

[21] Vanhove T, Kuypers D, Kathleen CJ, Evenepoel P, Meijers B, Naesens M (2013). Reasons for dose reduction of mycophnolate mofetil during the first year after renal transplantation and its impact on graft outcom. Transpl Int.

[22] Ushigome H, Okamoto M, Koshino K, Nobori S, Okajima H, Masuzawa N (2010). Findings of graft biopsy specimens within 90 days after ABO blood group incompatible living donor kidney transplantation compared with ABO-identical and non-identical transplantation. Clin Transpl.

[23] Le Meur Y, Büchler M, Thierry A, Caillard S, Villemain F, Lavaud S (2007). Individualized mycophnolate mofetil dosing based on drug exposure significantly improves patient outcomes after renal transplantation. Am J Transplant.

[24] Kuypers DR, de Jonge H, Naesens M, de Loor H, Halewijck E, Dekens M (2008). Current target ranges of mycophenolic acid exposure and drug-related adverse events: a 5-year, open-label, prospective, clinical follow-up study in renal allograft recipients. Clin Ther.

[25] de Klerk M, Kal-van Gestel JA, Haase-Kromwijk BJ, Claas FH, Weimar W, Living Donor Kidney Exchange Program (2011). Eight years of outcomes of the Dutch living kidney exchange program. Clin Transpl.

[26] Lonze BE, Bae S, Kraus ES, Holechek MJ, King KE, Alachkar N (2017). Outcomes and risk stratification for late antibody-mediated rejection in recipients of ABO-incompatible kidney transplants: a retrospective study. Transplant Int.

[27] Inui M, Miyazato T, Furusawa M, Okumi M, Omoto K, Ishida H (2016). Successful kidney transplantation after stepwise desensitization using rituximab and bortezomib in a highly HLA-sensitized and ABO incompatible high titer patient. Clin Transplant Direct.

